# Influence of diffusion on space-charge-limited current measurements in organic semiconductors

**DOI:** 10.3762/bjnano.4.18

**Published:** 2013-03-11

**Authors:** Thomas Kirchartz

**Affiliations:** 1 Department of Physics and Centre for Plastic Electronics, Imperial College London, South Kensington Campus SW7 2AZ, United Kingdom

**Keywords:** current–voltage curves, electron-only device, drift–diffusion, mobility, simulation, traps

## Abstract

Numerical simulations of current–voltage curves in electron-only devices are used to discuss the influence of charged defects on the information derived from fitting space-charge-limited current models to the data. Charged, acceptor-like defects lead to barriers impeding the flow of electrons in electron-only devices and therefore lead to a reduced current that is similar to the situation where the device has a built-in voltage. This reduced current will lead to an underestimation of the mobilities and an overestimation of characteristic tail slopes if analytical equations are used to analyze the data. Correcting for the barrier created by the charged defects can, however, be a successful way to still be able to obtain reasonably accurate mobility values.

## Introduction

A frequently used method to analyze charge carrier transport in organic semiconductors is based on space-charge-limited current measurements performed on single carrier devices [1–14]. These devices consist of two contacts that are either both electron-injecting or both hole-injecting, meaning that the current–voltage curve of these devices is not determined by the recombination of electrons and holes in the volume of the device [15] but instead by the mobility and concentration of carriers and the electric field in the device. If a device with two electron injecting contacts were doped to be sufficiently n-type that the electron concentration were determined by the doping and not by the injected charges in a certain range of voltages, the current–voltage curve in that range would be essentially ohmic, and the conductivity of the system would depend on mobility and electron concentration [16–17]. If the electron-only device were, however, undoped and the injection at the contacts efficient, the current density *J* would to a first approximation not depend on the equilibrium electron concentration anymore. Instead *J* would just depend on the mobility μ, which is typically the only unknown parameter, as well as the voltage *V*, the device thickness *d* and the permittivity ε = ε_0_ε_r_ and would ideally follow the Mott–Gurney law [18–19]

[1]
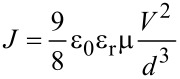


Here ε_0_ is the vacuum permittivity and *ε*_r_ is the relative permittivity. The Mott–Gurney law is frequently used to determine the mobility of organic semiconductors used for light emitting diodes and solar cells. However, its derivation uses three assumptions that are often not applicable in organic semiconductors, namely that the device is trap-free, that diffusion is negligible and that the electric field at the injecting contact is zero. All three assumptions are in general not correct, in particular the assumption that there are no charged defects in organic semiconductors [20–26]. While there have been numerous attempts to develop models to take traps in unipolar devices into account [27–31], nearly all of them still rely on drift as the only transport mechanism. However, traps will often lead to a situation where diffusion currents cannot be neglected anymore, which makes analytical approximations for this situation problematic [7,10,13].

In this article numerical simulations are used to show how the presence of traps leads to deviations from the analytical equations typically used to analyze single-carrier current–voltage curves. I will focus on acceptor-like traps in electron-only devices, i.e., situations where the traps are negatively charged when below the Fermi level. These negative charges will behave like p-type dopants and create a barrier for electrons. The electrons first have to diffuse over the barrier created by the negatively charged defects before they drift to the other contact. This barrier leads to an exponential increase of current with voltage for low voltages similar to the situation in a bipolar diode with a nonzero built-in voltage. To understand the influence of the trap-induced barrier on the interpretation of current–voltage curves, current–voltage curves are simulated for different concentrations of traps using a drift–diffusion solver, and then analytical equations are fitted to the simulated curves to compare the apparent mobility and density of states derived from the fit to the ones that were used as input for the drift–diffusion simulation. The results of the simulations show that using analytical equations in electron-only devices with substantial concentrations of negatively charged defects can result in strongly underestimated mobilities and overestimated width of the exponentially decaying density of states.

## Details of the simulation

The simulations are performed by using a commercial device simulator called Advanced Semiconductor Analysis (ASA) that was developed by the group of M. Zeman at the TU Delft (Netherlands) [32–33]. The software solves the Poisson equation

[2]
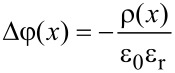


and the continuity equations for the electrons and holes

[3]
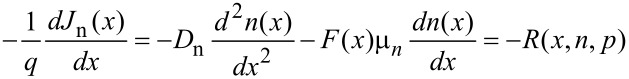


[4]
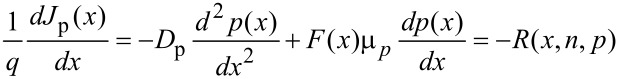


Here, *q* is the elementary charge, φ is the electrical potential, ρ is the space charge, *x* the spatial coordinate, *n* and *p* the free electron and hole concentrations, *F* the electric field, *D*_n,p_ the diffusion constant, and *J*_n_ and *J*_p_ are the electron and the hole current. Note that the classical Einstein relation (*D*_n,p_ = *kT*/*q* μ_n,p_) is used to connect the diffusion constant and the mobility. The Einstein relation is applicable even in disordered semiconductors if the continuity equations are expressed in terms of free carriers, as has been done here. The occupation of the traps follows Shockley–Read–Hall statistics and is described in detail in [34–36]. I use a Gaussian distribution of traps

[5]
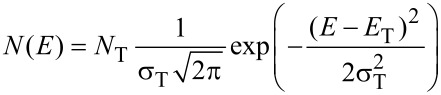


with a peak energy *E*_T_ at midgap and a width of σ_Τ_ = 100 meV similar to the values used by Nicolai et al. [13]. The total trap concentration *N*_T_ was varied in the simulations. It is assumed that the defects are acceptor-like defects, i.e., that the defect is negatively charged if occupied with an electron and neutral when empty. The opposite situation would be a donor-like effect that is positively charged when empty (occupied by a hole) and neutral when occupied with an electron. The rationale of using acceptor-like defects is that many organic semiconductors are known to be p-type, i.e., to have acceptor like defects, and to show improved transport after compensation of the p-type dopants with n-type dopants [8,23,26,37–38].

Because organic semiconductors are generally disordered materials, it is important to investigate the effects of energetic disorder on the results of our simulations. In order to take the effect of disorder into account, a multiple trapping model together with exponential band tails is used in some simulations (Figure 3 and Figure 5, see below). The energy-dependent densities of states *N*_CBT_ and *N*_VBT_ of these tails follow

[6]
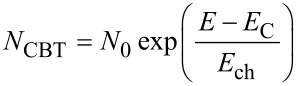


for the conduction-band tail and

[7]
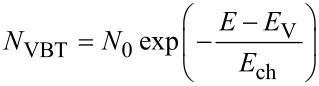


for the valence-band tail. Here, *N*_0_ is a prefactor with the unit 1/(cm^3^·eV) and defines the concentration of tail states per volume and energy interval at the conduction band and valence band edge *E*_C_ and *E*_V_. The conduction band tail is assumed to consist of acceptor-like defects and the valence band tail of donor-like defects [34,39]. It is assumed that the mobility of electrons is zero below the conduction band edge and has a constant value above, i.e., the influence from Poole–Frenkel type effects is neglected for simplicity. The slope of the tails is given by *E*_ch_ and assumed to be the same for the conduction and valence band in all cases. The boundary conditions at the contacts are defined by keeping the distance between conduction band edge and Fermi level constant at 0.1 eV for both contacts. Both contacts have high (10^5^ cm/s) recombination velocities for electrons and holes (cf. [40] for an exact definition of the boundary conditions).

## Results and Discussion

### The influence of diffusion on the current–voltage curves

**Figure 1:**
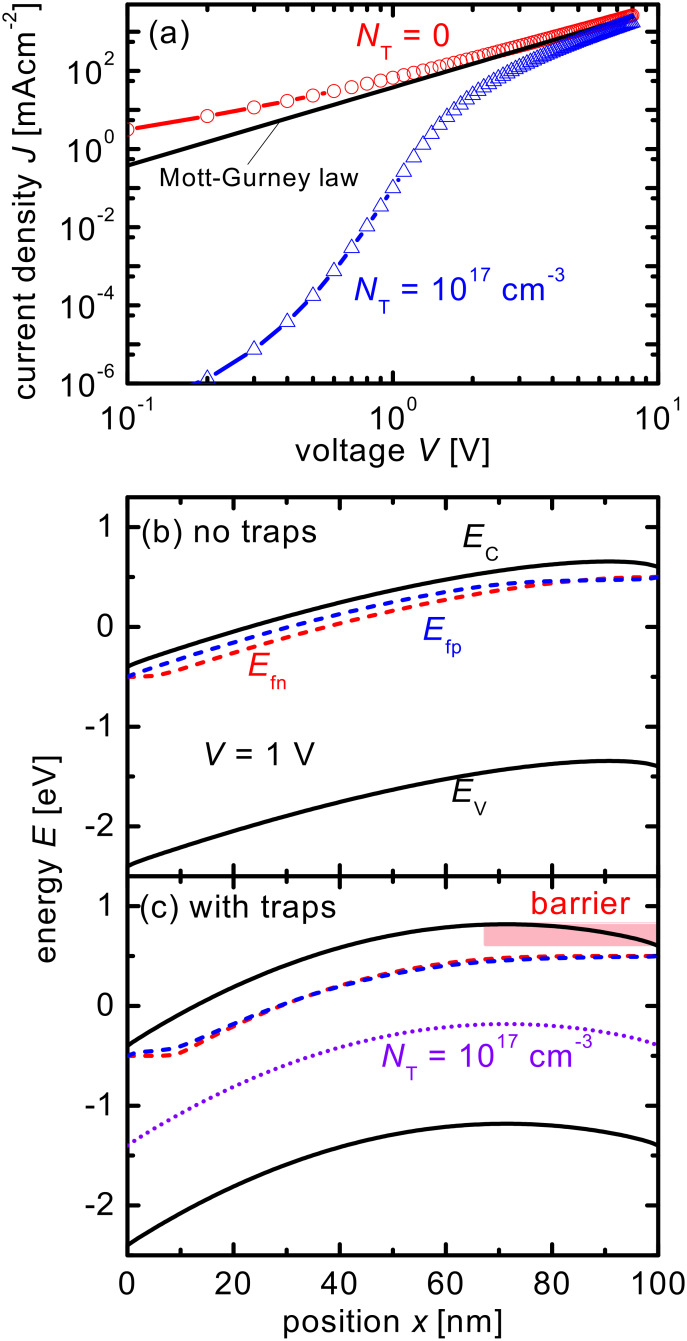
(a) Current–voltage curves of a device with and without charged acceptor-like defects with a total concentration *N*_T_ = 10^17^ cm^−3^ and a Gaussian width of σ = 100 meV are compared to the Mott–Gurney law (Equation 1). Band diagram of the device (b) without charged defects and (c) with charged defects. The acceptor-like defects in (c) create a barrier (indicated with a light red background) due to their negative charge. The diffusion of electrons up the barrier causes the reduced current in (a).

To understand the effect of charged defects on the current–voltage curve of electron-only devices it is most instructive to compare the simulated current–voltage curves with band diagrams. Figure 1 compares the situation with and without acceptor-like traps and depicts both the current–voltage curves as well as the band diagrams at *V* = 1 V forward bias (electrons are injected on the right and extracted on the left). Table 1 gives the parameters used for the simulations. Both simulated current–voltage curves are compared to the analytical equation (Mott–Gurney law) given by Equation 1. Interestingly, already the simulation without any traps is only well reproduced by the Mott–Gurney law at higher voltages, while its slope tends to be more ohmic (~*V*) than space-charge-limited (~*V*^2^) at lower voltages. This phenomenon has been described in the past [41] and is related to the movement of the virtual cathode (the point of zero electric field) as a function of voltage. Figure 1b shows that the point of zero field is close to but not at the cathode, even in the cases without traps. In the derivation of the Mott–Gurney law it is assumed that the point of zero electric field is fixed at the actual cathode, which has previously been shown to be incorrect [10].

**Table 1:** All the parameters used in the simulations, if not stated otherwise. For the definition of the capture coefficients see Figure 2 in [39]. The contact barrier is the distance between the Fermi level and conduction band edge at both contacts. This value is kept constant for all simulations except for the one with *V*_bi_ = 1 V in Figure 2, where the contact barrier at the cathode (*x* = *d*) is 0.1 eV and the contact barrier at the anode (*x* = 0) is 1.1 eV. The relative permittivity used in all simulations is ε_r_ = 3.8 and the capture coefficient for the Gaussian defect is 10^−10^ cm^3^·s^−1^ for electrons and holes.

		no tails	with tails

figure no.		1–4	3,5

mobility	μ_0_ [cm^2^/Vs]	10^−4^	10^−3^
effective density of states	*N*_C_ = *N*_V_ [cm^−3^]	10^20^	10^20^
density of tail states	*N*_0_ [eV^−1^cm^−3^]	0	10^20^
characteristic tail slope	*E*_ch_ [meV]	0	variable or 50

capture coefficients (tails)	β_n_^+^ [cm^3^·s^−1^]	0	10^−12^
	β_p_^0^ [cm^3^·s^−1^]	0	10^−10^
	β_p_^−^ [cm^3^·s^−1^]	0	10^−12^
	β_n_^0^ [cm^3^·s^−1^]	0	10^−10^

band gap	*E*_g_ [eV]	2.0	2.0
thickness	*d* [nm]	100	100
surface recombination velocity	*S* [cm/s]	10^5^	10^5^
contact barrier	φ_b_ [meV]	0.1	0.1

The most obvious effect, however, is that the current–voltage curve of the device with traps is strongly reduced at low voltages relative to both the simulation without traps and the Mott–Gurney law. This is due to the barrier formed by the charged defects as highlighted by the light red background in the band diagram in Figure 1c. The traps are essentially always below the quasi Fermi levels for electrons and holes and will therefore be occupied with electrons. The space charge of the electrons on the traps creates an electrostatic barrier (highlighted in red) close to the injecting contact that impedes the flow of electrons from the injecting cathode at *x* = *d* towards the electron extracting contact at *x* = 0. The existence of this barrier means that more voltage has to be applied to achieve the same current flow than without the barrier. Thus, the current at a given voltage is reduced as seen in Figure 1a. In this context, it is not important where the energy level of the defect is; as long as it is an acceptor-like defect that is energetically below the quasi Fermi levels for electrons and holes, the behavior will be the same. However, when the Gaussian distribution of defects is close to the quasi Fermi level at a given voltage only part of the distribution will be occupied by electrons and therefore only part of the distribution will contribute to the band bending.

**Figure 2:**
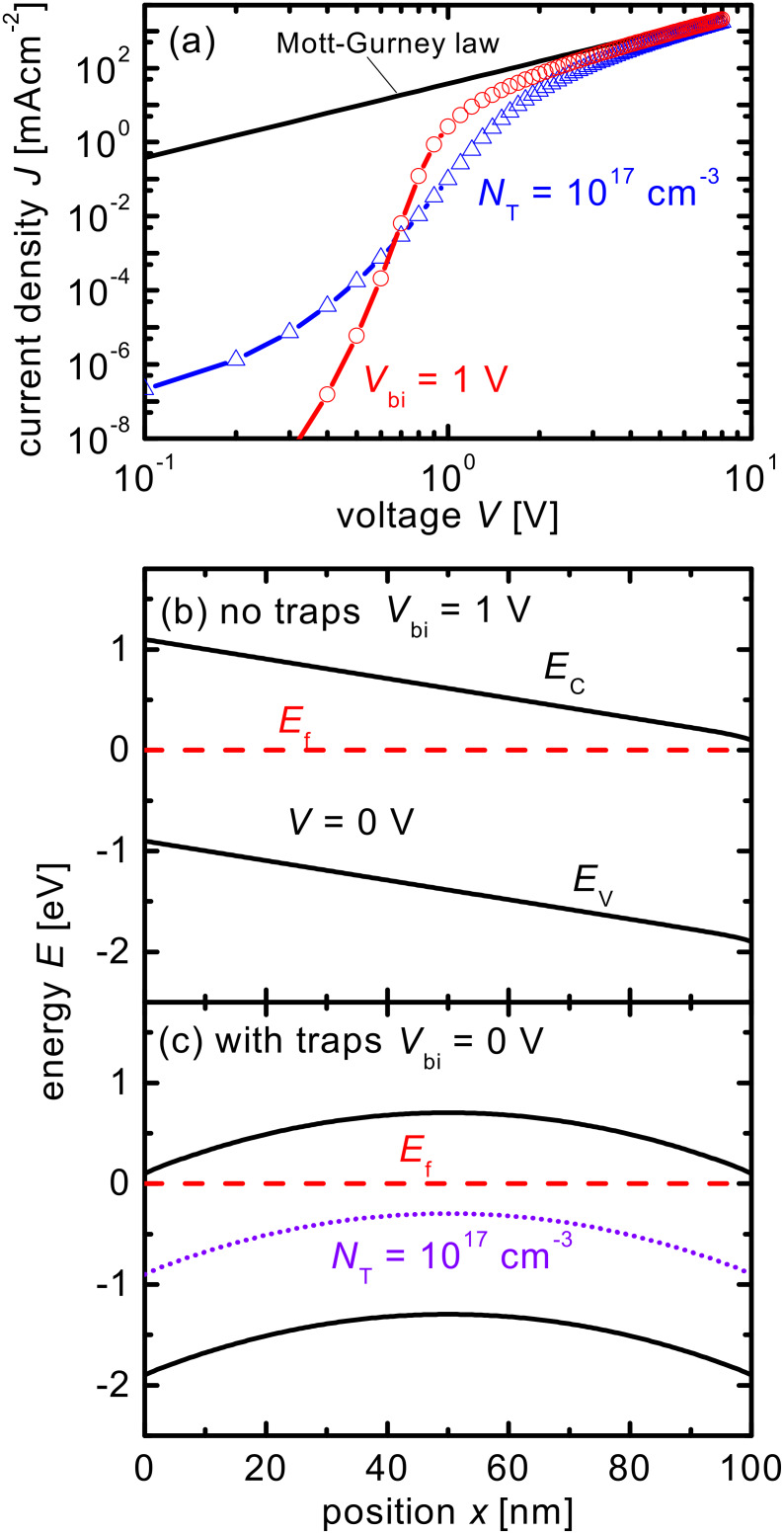
(a) Current–voltage curves of a device with charged acceptor-like defects and a built-in voltage *V*_bi_ = 0 V (as in Figure 1a) and a device with no defects but a built-in voltage *V*_bi_ = 1 V are compared to the Mott–Gurney law (Equation 1). Band diagram of the device (b) without charged defects and *V*_bi_ = 1 V and (c) with charged defects and *V*_bi_ = 0 V. Both band diagrams are depicted at short circuit.

Apart from acceptor-like defects, also nonzero built-in voltages can lead to a situation where diffusion currents dominate the current–voltage curve at low voltages. Figure 2a shows the current–voltage curves of a device with a built-in voltage *V*_bi_ = 1 V as compared to the current–voltage curve of the device with traps that has been shown already in Figure 1. The built-in voltage is created by setting the distance *E*_C_ − *E*_F_ = 1.1 eV at the extracting contact while keeping *E*_C_ − *E*_F_ = 0.1 eV at the injecting contact. In both cases, the qualitative behavior is similar with a strongly reduced current at low voltages. The band diagrams in Figure 2b and Figure 2c are now depicted at short circuit and show that the shape of the barrier is completely different in both cases but the height correlates with the amount of reduction in current. The barrier in the case of the asymmetric contacts is 1 V and leads to a stronger reduction in current at low voltages than the smaller barrier in the case of the symmetric contacts with traps.

### The determination of mobilities using analytical equations

This comparison between two types of barriers is relevant because the built-in voltage is often not known precisely [11] and it is customary to correct for its influence by using *V* − *V*_bi_ as the voltage axis [4–5]. At first, this seems like a problem, because with the built-in voltage as a free parameter, it might be possible to erroneously assign the influence of a trap to a higher *V*_bi_. To investigate that problem drift–diffusion simulations were performed for a device with *V*_bi_ = 0 but with a varying concentration of trap states, and the resulting current–voltage curves were fitted to the Murgatroyd equation [19]

**Figure 3:**
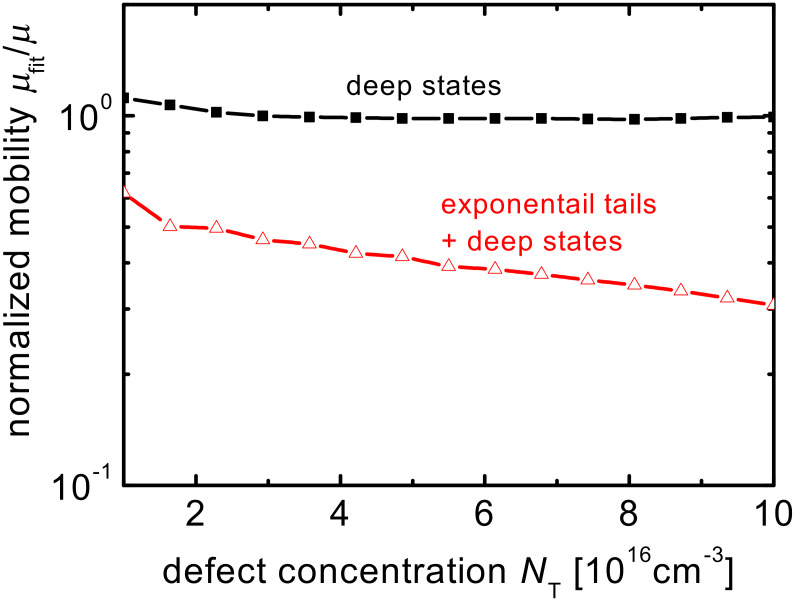
Normalized mobility obtained by fitting the Murgatroyd equation to the simulated current–voltage curves of electron-only devices as a function of the concentration of midgap defects. The fitted mobility is normalized to the actual mobility that is used as an input parameter in the simulation and the built-in voltage in Equation 8 is a free parameter. The actual built-in voltage used as input for the simulated current–voltage curves is zero. The comparison between fitted and actual mobility is done for two situations, one (lines + filled squares) with only a variable concentration of midgap defects and one (lines + open triangles) with the same variable concentration of defects and additional exponential tails.

[8]
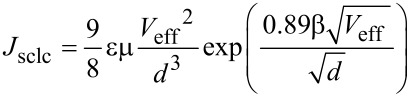


that is frequently used to determine the mobility of organic semiconductors [9,42]. Here the parameter β controls the field dependence of the current that is meant to describe the Poole–Frenkel effect. However, in practice the factor β may be affected by the influence of traps and trap-induced diffusion currents. For the simulations shown in Figure 3 (see below), the voltage *V*_eff_ is defined as *V*_eff_ = *V* − *V*_bi_ with *V*_bi_ being a fit parameter to understand the situation when the influence of traps is erroneously attributed to an increased *V*_bi_.

Figure 3 shows the mobility resulting from the fit of Equation 8 to the numerical simulation normalized to the value that was used as input for the simulation (μ = μ_n_ = μ_p_ = 10^−4^ cm^2^/Vs). Note that the results hardly change when changing the mobility in the range typical for organic semiconductors, therefore the absolute value of the mobility is of limited importance in this context. The simulated device is an electron-only device with a thickness of 100 nm. See Table 1 for the parameters used in the simulation. The first data set (line + filled squares) assumes that there are no exponential tails and it leads to a nearly perfect reproduction of the actual mobility. Thus, although the correction for *V*_bi_ is completely nonphysical the fitted mobilities are very close to the ones used as input for the simulations. Even in a case where there are exponential tails in addition to the variable concentration of midgap defects (line + open triangles), the correction for *V*_bi_ leads to a reasonably well approximated value for the mobility. Although there is a mismatch between fitted mobility and real mobility, this mismatch is only weakly dependent on the concentration of traps and not unexpected given that the Murgatroyd equation was not developed to deal with exponential tails. Note that in the case with exponential tails, the mobility is an effective value calculated as

[9]
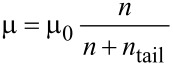


where μ_0_ is the band mobility of the free electrons, *n* is the concentration of free electrons and *n*_tail_ the concentration of electrons trapped in the conduction band tail. The voltage at which the carrier concentrations are evaluated was arbitrarily chosen as *V* = 1 V. This effective value takes into account that only a part of the electrons is able to move and another part is trapped in the shallow tail states.

**Figure 4:**
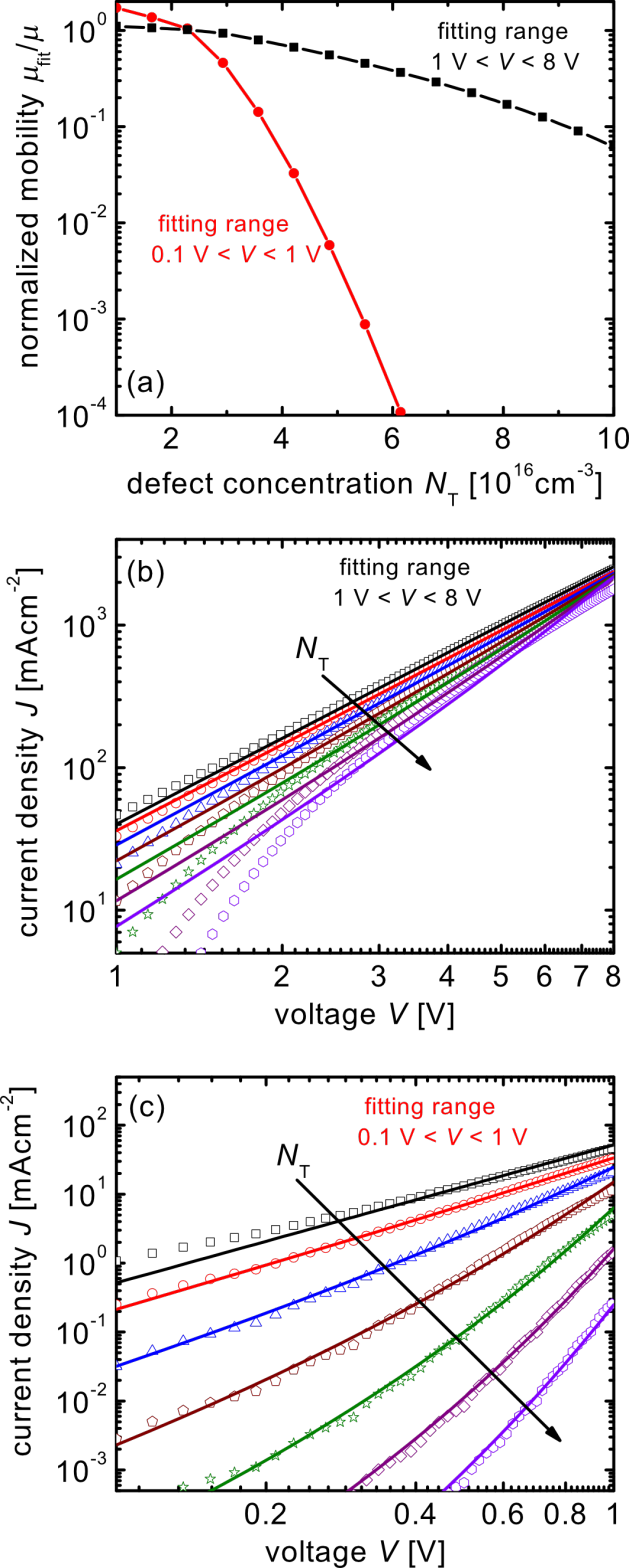
Normalized mobility as a function of defect concentration if the built-in voltage in Equation 8 is set to 0. Now, no correction for the barrier created by the charged defects is done and the mobility is underestimated at high trap concentrations. Because the current–voltage curve is affected by the traps mostly at low voltages, the mobility resulting from the fit will depend strongly on the range used for fitting. Reasonable fit quality is either reached if the fit is done for higher voltages *V* > 1 V or for lower *V* < 1 V. (b) and (c) show the fits (lines) to the drift–diffusion simulations (symbols) for the two different fitting ranges. Every second datapoint in (a) is presented in (b) and (c).

If the built-in voltage in the fit of Equation 8 is set to the correct value of zero, obtaining the correct mobility using the Murgatroyd equation becomes impossible at high trap concentrations. Figure 4a shows the normalized mobility as a function of trap concentration for two different voltage ranges in which the Murgatroyd equation is fitted to the simulated data. Because the analytical equation neglects the predominant effect of diffusion at low voltages and high defect concentrations, the fits predict mobilities that are a strong function of defect concentration and that change dramatically depending on the voltage range that is analyzed. There are two voltage ranges in which the Murgatroyd equation can lead to a reasonable fit to the data. For high voltages *V* > 1 V (see Figure 4b), the influence of space charge on the current–voltage curves is relatively weak and the Murgatroyd equation gives a good fit with a moderate uncertainty in mobility (one order of magnitude for a trap density *N*_T_ = 10^17^ cm^−3^). For low voltages *V* < 1 V, the exponential dependence of current density on 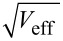 can lead to a good fit by using high values of β and extremely low values of the mobility μ as shown in Figure 4c. In an ideal case with *V*_bi_ = 0 and a negligible series resistance it would not be possible to fit the whole range of voltages from around 0.1 V to *V* >> 1 V well by using the Murgatroyd equation. However, as shown in Supporting Information File 1, when correcting for series resistances the shape of a fit using the Murgatroyd equation can look very similar to a simulation with charged defects.

The result that good fits are possible if data is corrected for a voltage barrier means that the physical situations of having an actual built-in voltage (see Figure 2b) and having a concentration of traps that leads to a barrier for majority-carrier transport (Figure 2c) are similar in their effect on the current–voltage curve. In both cases, the device behaves like a series connection of a diode-like element (the barrier) and a space-charge-limited current regime. Correcting the voltage axis for the effective barrier will in both cases allow us to isolate the contribution from the space-charge-limited current regime. In this context it may therefore be more important to understand that there may be different origins of voltage barriers (the contacts or the properties of the bulk), than to actually use a different strategy to analyze the data in terms of mobilities. In addition, the results suggest that any data analysis based on interpreting the diffusion currents at low forward voltages in order to determine properties of traps [7,13] needs to exercise care in attributing the diffusion to bulk or contact effects (see Figure S1 in Supporting Information File 1). To discriminate between barriers due to bulk or contact effects, forward and reverse bias current–voltage curves should be analyzed. Because bulk effects would be symmetric while contact effects are not (see Figure S2 in Supporting Information File 1), the reverse-bias current–voltage curve may be used to determine the built-in voltage. An alternative method could be to measure the capacitance of the single-carrier device and analyze it as suggested by van Mensfoort and Coehoorn [43].

### The determination of characteristic tail slopes from current–voltage curves

Although space-charge-limited current measurements are most frequently used to measure mobilities in organic semiconductors, there have been attempts to use the slope of the current–voltage curve on a log–log plot as a measure of the density of localized states. The density of localized states is an important property of the material, affecting both transport [44–45] and recombination [36,39,46–57], and one that can change as a function of device processing and during device degradation [55]. Usually, an exponential tail of states is assumed that is characterized by a characteristic energy *E*_ch_ as defined in Equation 6 and Equation 7. Then, analytical equations are used, as for instance the one by Mark and Helfrich [28]. While the analytical approximations usually vary in their prefactors, they agree on the proportionality between current and voltage that follows [1,27,29,31,58]

[10]
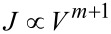


where

[11]
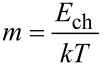


**Figure 5:**
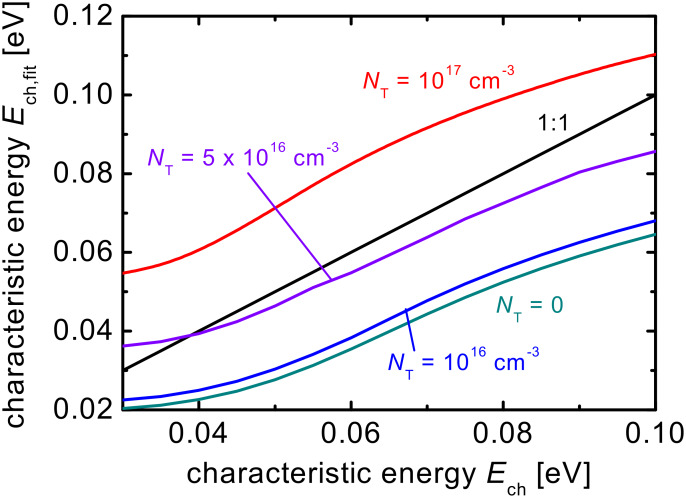
Comparison of the characteristic energy *E*_ch,fit_ obtained by fitting Equation 10 to simulated current–voltage curves of electron-only devices assuming the characteristic energy *E*_ch_ of the tail states that was used as input in the simulation. A high concentration of defects (*N*_T_ = 10^17^ cm^−3^) leads to a reduction of the current as shown in Figure 1a, which implies a much steeper slope of the curve in a certain voltage range. This will then be interpreted as a broader tail slope by Equation 10. If the concentration of defects is zero or low, Equation 10 will however underestimate the tail slope. The voltage range for the fits is 1 V < *V* < 8 V for the case with *N*_T_ = 10^17^ cm^−3^ and 0.1 V < *V* < 8 V for all other cases.

This approximation, however, still requires diffusion currents to be negligible. Thus, it is useful to look at the relation between the tail slope that is used as input for the model and the tail slope determined from a fit of Equation 10 to the current–voltage curve. Figure 5 depicts this comparison for different values of the concentration of midgap defects. The more defects the system contains, the more diffusion will be relevant. Diffusion currents however, as shown in Figure 1a and Figure 2a, lead to a steeper increase of current with voltage than predicted by the Mott–Gurney law. This steep increase of current will be interpreted as a high value of *m* and therefore *E*_ch_, when fitting Equation 10 to the data. Thus, for higher concentrations of charged acceptor-like defects, the tail slope determined by the fit (see Supporting Information File 1 for the fits and simulations) will overestimate the real tail slope. However, for intermediate and low concentrations of charged defects, Equation 10 actually underestimates the tail slope showing that even in intrinsic devices, the approximations of negligible diffusion and zero field at the cathode are not correct and lead to deviations between a full numerical model and the analytical approximation.

## Conclusion

It has been shown that charged acceptor-like defects lead to barriers in electron-only devices that increase the relevance of diffusion currents. Because diffusion currents are always neglected when analytical equations are used to analyze either the mobility μ or the slope *E*_ch_ of an exponential band tail from single carrier devices, these defects will lead to incorrect results. Interestingly, when the voltage is corrected for a current-independent barrier, as is often done in practice to correct for a nonzero built-in voltage, the error in the mobility determined by fitting of an analytical equation and the one used as input for the simulation is low. In contrast, when the data is uncorrected, the mobility will both depend strongly on the voltage range of the fit and the concentration of charged defects. For instance, defect concentrations of *N*_T_ = 10^17^ cm^−3^ can lead to a fitted mobility that is several orders of magnitude smaller than the actual mobility. In case of the determination of the tail slope from the current–voltage curve of electron-only devices, a higher concentration of acceptor-like defects will lead to an overestimation and a lower concentration to an underestimation of the actual tail slope. Here it is important to note that even without any charged defects, the tail slope is not particularly well reproduced by the typically used analytical equations.

## Supporting Information

Figure S1 shows the similarity between current–voltage curves affected by (i) a nonzero built-in voltage and (ii) space charge due to charged defects. The effect of built-in voltage on forward- and reverse-bias current–voltage curves is shown in Figure S2. Figure S3 discusses the effect of series resistances of fitting current–voltage curves with the Murgatroyd equation. Figures S4 to Figure S6 are the fits used to create Figure 5.

File 1 Additional simulations.

## References

[1] Beiley Z M, Hoke E T, Noriega R, Dacuña J, Burkhard G F, Bartelt J A, Salleo A, Toney M F, McGehee M D (2011). Adv Energy Mater.

[2] Nicolai H T, Wetzelaer G A H, Kuik M, Kronemeijer A J, de Boer B, Blom P W M (2010). Appl Phys Lett.

[3] Lenes M, Shelton S W, Sieval A B, Kronholm D F, Hummelen J C, Blom P W M (2009). Adv Funct Mater.

[4] Lenes M, Morana M, Brabec C J, Blom P W M (2009). Adv Funct Mater.

[5] Mihailetchi V D, Koster L J A, Blom P W M, Melzer C, de Boer B, van Duren J K J, Janssen R A J (2005). Adv Funct Mater.

[6] Mihailetchi V D, van Duren J K J, Blom P W M, Hummelen J C, Janssen R A J, Kroon J M, Rispens M T, Verhees W J H, Wienk M M (2003). Adv Funct Mater.

[7] Nicolai H T, Mandoc M M, Blom P W M (2011). Phys Rev B.

[8] Lu M T, Nicolai H T, Wetzelaer G-J A H, Blom P W M (2011). J Polym Sci, Part B: Polym Phys.

[9] Azimi H, Senes A, Scharber M C, Hingerl K, Brabec C J (2011). Adv Energy Mater.

[10] Dacuña J, Salleo A (2011). Phys Rev B.

[11] Dacuña J, Xie W, Salleo A (2012). Phys Rev B.

[12] Kuik M, Wetzelaer G-J A H, Laddé J G, Nicolai H T, Wildeman J, Sweelssen J, Blom P W M (2011). Adv Funct Mater.

[13] Nicolai H T, Kuik M, Wetzelaer G A H, de Boer B, Campbell C, Risko C, Brédas J L, Blom P W M (2012). Nat Mater.

[14] Faist M A, Shoaee S, Tuladhar S, Dibb G F A, Foster S, Gong W, Kirchartz T, Bradley D D C, Durrant J R, Nelson J (2013). Adv Energy Mater.

[15] Wetzelaer G A H, Kuik M, Lenes M, Blom P W M (2011). Appl Phys Lett.

[16] Jain S C, Geens W, Mehra A, Kumar V, Aernouts T, Poortmans J, Mertens R, Willander M (2001). J Appl Phys.

[17] Zhang X-G, Pantelides S T (2012). Phys Rev Lett.

[18] Mott N F, Gurney R W (1940). Electronic Processes in Ionic Crystals.

[19] Murgatroyd P N (1970). J Phys D: Appl Phys.

[20] Gregg B A, Gledhill S E, Scott B (2006). J Appl Phys.

[21] Liang Z, Nardes A, Wang D, Berry J J, Gregg B A (2009). Chem Mater.

[22] Hains A W, Liang Z, Woodhouse M A, Gregg B A (2010). Chem Rev.

[23] Liang Z, Gregg B A (2012). Adv Mater.

[24] Liang Z, Nardes A M, van de Lagemaat J, Gregg B A (2012). Adv Funct Mater.

[25] Khelifi S, Decock K, Lauwaert J, Vrielinck H, Spoltore D, Piersimoni F, Manca J, Belghachi A, Burgelman M (2011). J Appl Phys.

[26] Bisquert J, Garcia-Belmonte G (2011). J Phys Chem Lett.

[27] Lampert M A (1956). Phys Rev.

[28] Mark P, Helfrich W (1962). J Appl Phys.

[29] Rose A (1955). Phys Rev.

[30] Woellner C F, Freire J A (2011). J Chem Phys.

[31] Campbell A J, Bradley D D C, Lidzey D G (1997). J Appl Phys.

[32] Zeman M, Krc J (2008). J Mater Res.

[33] Pieters B E, Stiebig H, Zeman M, van Swaaij R A C M M (2009). J Appl Phys.

[34] Pieters B E (2008). Characterization of Thin-Film Silicon Materials and Solar Cells through Numerical Modelling.

[35] Pieters B E, Decock K, Burgelman M, Stangl R, Kirchartz T (2011). One-Dimensional Electro-Optical Simulations of Thin-Film Solar Cells. Advanced Characterization Techniques for Thin Film Solar Cells.

[36] Kirchartz T, Nelson J (2012). Phys Rev B.

[37] Zhang Y, de Boer B, Blom P W M (2010). Phys Rev B.

[38] Lu M, Nicolai H T, Wetzelaer G-J A H, Blom P W M (2011). Appl Phys Lett.

[39] Kirchartz T, Pieters B E, Kirkpatrick J, Rau U, Nelson J (2011). Phys Rev B.

[40] Kirchartz T, Gong W, Hawks S A, Agostinelli T, MacKenzie R C I, Yang Y, Nelson J (2012). J Phys Chem C.

[41] Grinberg A A, Luryi S (1987). J Appl Phys.

[42] Mihailetchi V D, Xie H X, de Boer B, Koster L J A, Blom P W M (2006). Adv Funct Mater.

[43] van Mensfoort S L M, Coehoorn R (2008). Phys Rev Lett.

[44] Choulis S A, Nelson J, Kim Y, Poplavskyy D, Kreouzis T, Durrant J R, Bradley D D C (2003). Appl Phys Lett.

[45] Chatten A J, Tuladhar S M, Choulis S A, Bradley D D C, Nelson J (2005). J Mater Sci.

[46] Garcia-Belmonte G, Boix P P, Bisquert J, Lenes M, Bolink H J, La Rosa A, Filippone S, Martin N (2010). J Phys Chem Lett.

[47] Garcia-Belmonte G, Bisquert J (2010). Appl Phys Lett.

[48] Tachiya M, Seki K (2010). Phys Rev B.

[49] Nelson J (2003). Phys Rev B.

[50] MacKenzie R C I, Kirchartz T, Dibb G F A, Nelson J (2011). J Phys Chem C.

[51] Schafer S, Petersen A, Wagner T A, Kniprath R, Lingenfelser D, Zen A, Kirchartz T, Zimmermann B, Würfel U, Feng X J (2011). Phys Rev B.

[52] Street R A, Schoendorf M, Roy A, Lee J H (2010). Phys Rev B.

[53] Street R A, Song K W, Northrup J E, Cowan S (2011). Phys Rev B.

[54] Street R A (2011). Phys Rev B.

[55] Street R A, Krakaris A, Cowan S R (2012). Adv Funct Mater.

[56] MacKenzie R C I, Shuttle C G, Chabinyc M L, Nelson J (2012). Adv Energy Mater.

[57] Blakesley J C, Neher D (2011). Phys Rev B.

[58] Paasch G, Scheinert S (2009). J Appl Phys.

