# Supplementary material for: Influence of diffusion on space-charge-limited current measurements in organic semiconductors
Source: Beilstein J Nanotechnol. 2013 Mar 11;4:180–8. doi: 10.3762/bjnano.4.18 (PMC3628774; doi:10.3762/bjnano.4.18)
Supplement: File 1 — Additional simulations. [file Beilstein_J_Nanotechnol-04-180-s001.pdf]

Supporting Information

for

Influence of diffusion on space-charge-limited current measurements in organic semiconductors

Thomas Kirchartz*

Address: Department of Physics and Centre for Plastic Electronics, Imperial College London, South Kensington Campus SW7 2AZ, United Kingdom

Email: Thomas Kirchartz - t.kirchartz@imperial.ac.uk

* Corresponding author

Additional simulations

Figure S1 shows the similarity between current–voltage curves affected by (i) a nonzero built-in voltage and (ii) space charge due to charged defects. The effect of built-in voltage on forward and reverse bias current–voltage curves is shown in Figure S2. Figure S3 discusses the effect of series resistances of fitting current–voltage curves with the Murgatroyd equation. Figures S4 to Figure S6 are the fits used to create Figure 5.

Similarity between current–voltage curves affected by a nonzero built-in voltage and space charge

In the main paper it is claimed that the qualitative shape of the current–voltage curve in the case of charged defects and in the case of nonzero built-in voltages is similar. To show this point and discuss its consequences, I show that it is possible to obtain the same shape of the *forward* current–voltage curve with different values of the built-in voltage if the properties of the traps are changed at the same time. For this purpose, I simulated a single carrier device with V_{bi} = 0.2V and midgap traps (N_T = 5×10^{16} cm^{-3}) and then tried to fit it by assuming different values for V_{bi} ranging from 0 to 0.6 V as shown in Figure S1. In the whole range of V_{bi}s it is possible to fit the current–voltage curve quite nicely by slightly changing the parameters of the traps (see Figure S1b). This proves that the functional dependence of the effect of traps or a V_{bi} is very similar in forward bias. An uncertainty in the built-in voltage by 400 mV can lead to errors in the estimation of the trap depth of more than 600 meV and errors in the trap density of 30 %.

If, however, the reverse bias sweep is taken into account as well, the effect of the built-in voltage and the charged defects is substantially different. The charged defects will lead to a perfectly symmetric current–voltage curve, while a nonzero built-in voltage will lead to an asymmetry in current–voltage curves. In an electron-only device with a built-in voltage, if electrons are injected from the side where the distance to the Fermi-level conduction band edge is smaller (forward bias), current will be low at small bias because the built-in voltage has to be overcome first and then increase strongly before the current will become space-charge-limited and scale with V^2. If instead, electrons are injected from the side with the larger distance between Fermi level and conduction band edge, the current will be lower over the whole range of voltages but have a qualitatively similar shape. This is shown in Figure S2. Analyzing and using this effect might help to determine the properties of traps using single carrier devices with higher precision.

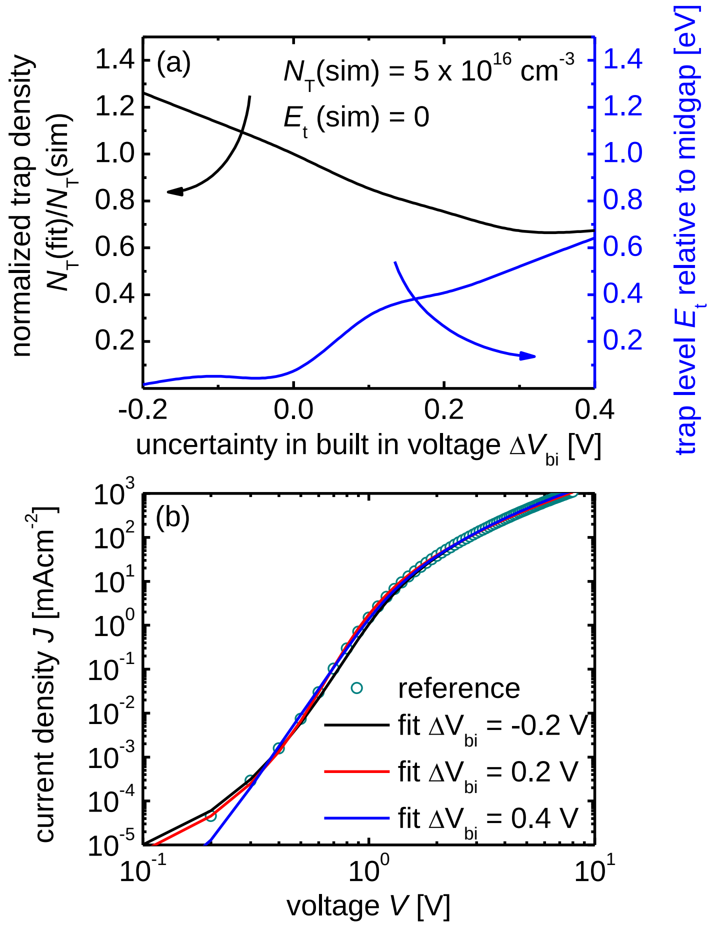

Figure S1: (a) Errors in trap parameters when the wrong built-in voltage is used for simulation as a function of the difference in built-in voltage between the simulation and the fit. (b) Some of the fits (solid lines) compared to the reference (open circles). In the whole range of V_{bi} shown here, the fit is excellent for currents higher than 10^{-4} mAcm^{-2}.

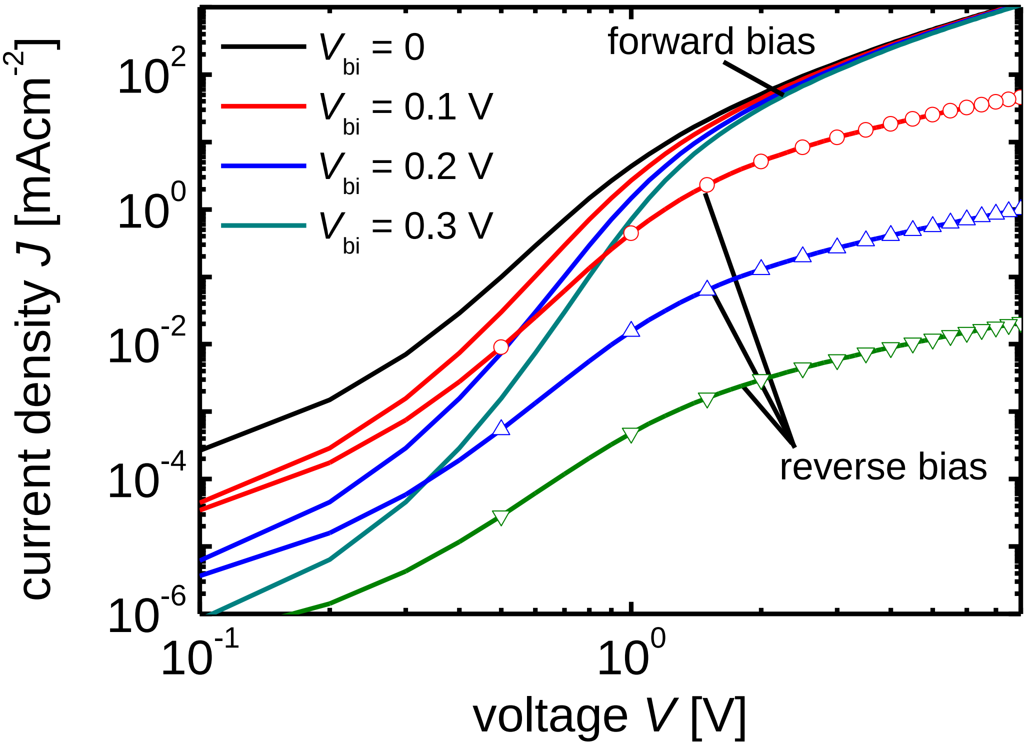

Figure S2: Comparison of the effect of a built-in voltage V_{bi} on the forward and reverse bias current–voltage curves in single carrier devices. For all simulations, the concentration of midgap traps was kept constant at $N_T = 5 \times 10^{16}$ cm^{-3}.

Murgatroyd equation in the presence of series resistances

The shape of the Murgatroyd equation is in principle substantially different from the shape of a current–voltage curve of a single carrier device with charged defects. Although the Murgatroyd equation can fit the low voltage regime and the high voltage regime well, it cannot fit both well at the same time. This might suggest that the likelihood of misinterpreting trap-limited conduction as a field-dependent mobility should be very low. However real devices have finite series resistances and fits are usually corrected for this series resistance. Especially if the series resistance is not known precisely (and varied to fit the data), excellent fits could be made with the Murgatroyd equation that would reproduce trap limited current–voltage curves very well. In Figure S3 we show simulations to illustrate this point. Figure S3a shows the resulting decrease in zero-field mobility if the Murgatroyd equation corrected for a variable or fixed series resistance is fitted to simulated curves with a fixed series resistance of 5 Ωcm^2. Figure S3b shows the corresponding fits (every second point in (a) is presented) for the case of a variable series resistance. The fits are all of high quality and the series resistance needed to fit the data is in all cases around 10 Ωcm^2. If the series resistance is kept fixed at 5 Ωcm^2 the quality of the fits as shown in Figure S3c is reduced, especially at high voltages.

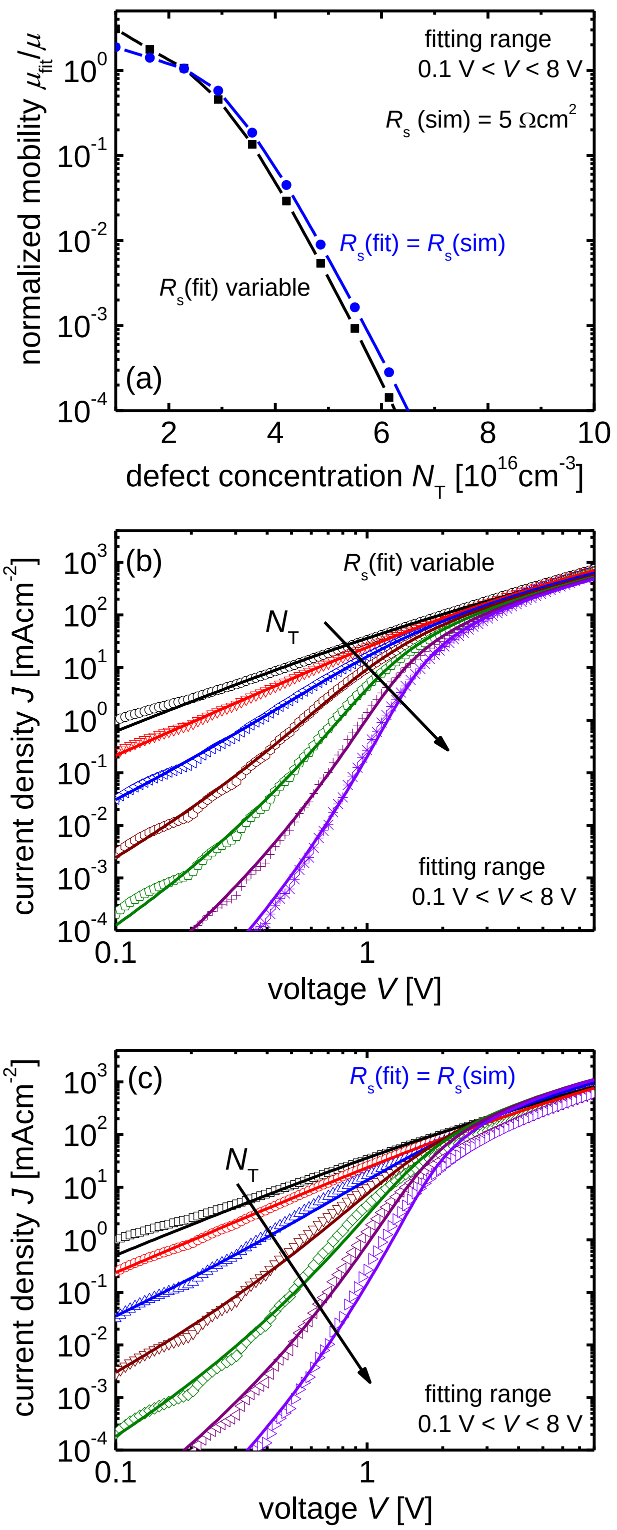

Figure S3: Normalized mobility as a function of defect concentration if the built-in voltage in Equation 8 is set to 0 but with a finite series resistance that is either fitted to the simulations or fixed to the value used in the simulations. In both cases, the mobility is drastically underestimated for moderate defect concentrations $N_T > 4 \times 10^{16}$ cm^{-3}. However, only in the case with variable mobility

the quality of the fits at high bias remains high for high defect concentrations as shown in (b). In the case of a fixed series resistance (c) high and low voltages cannot be both fitted with high accuracy.

Determination of the characteristic tail slope – fits

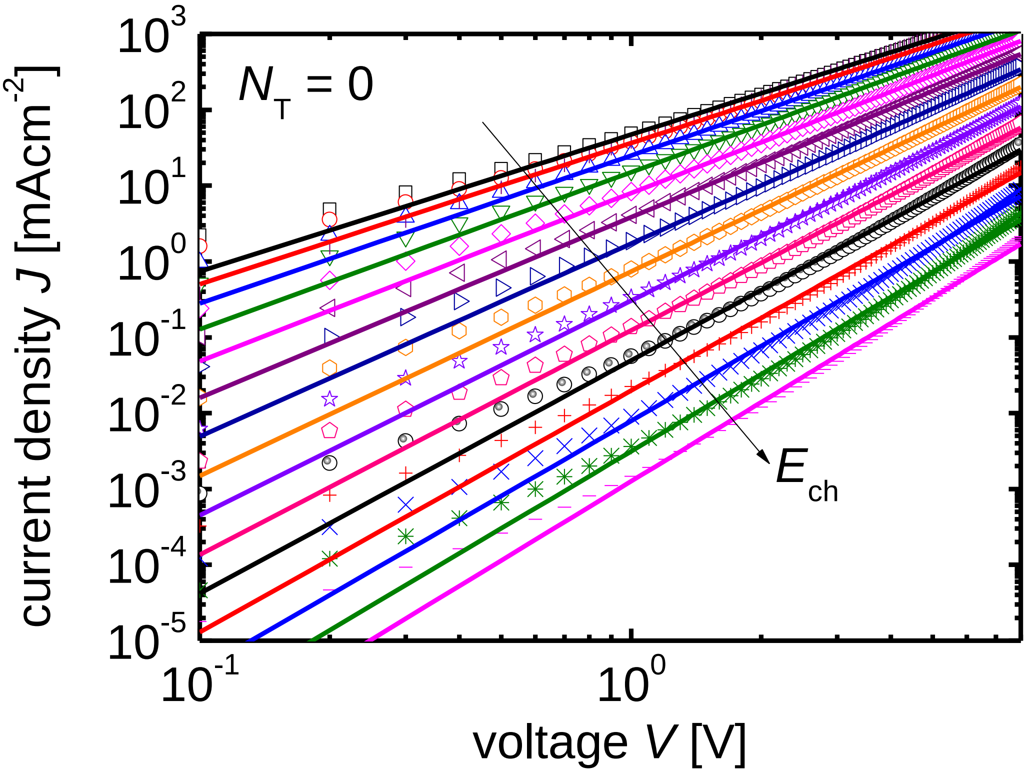

Figure S4: Fits for the trap-free case for different characteristic tail slopes E_{ch} from 30 meV to 100 meV. Symbols are the simulations with the drift-diffusion solver and lines are the fits with the Mark–Helfrich equation. The fits are done in the voltage range 0.1 V < V < 8 V.

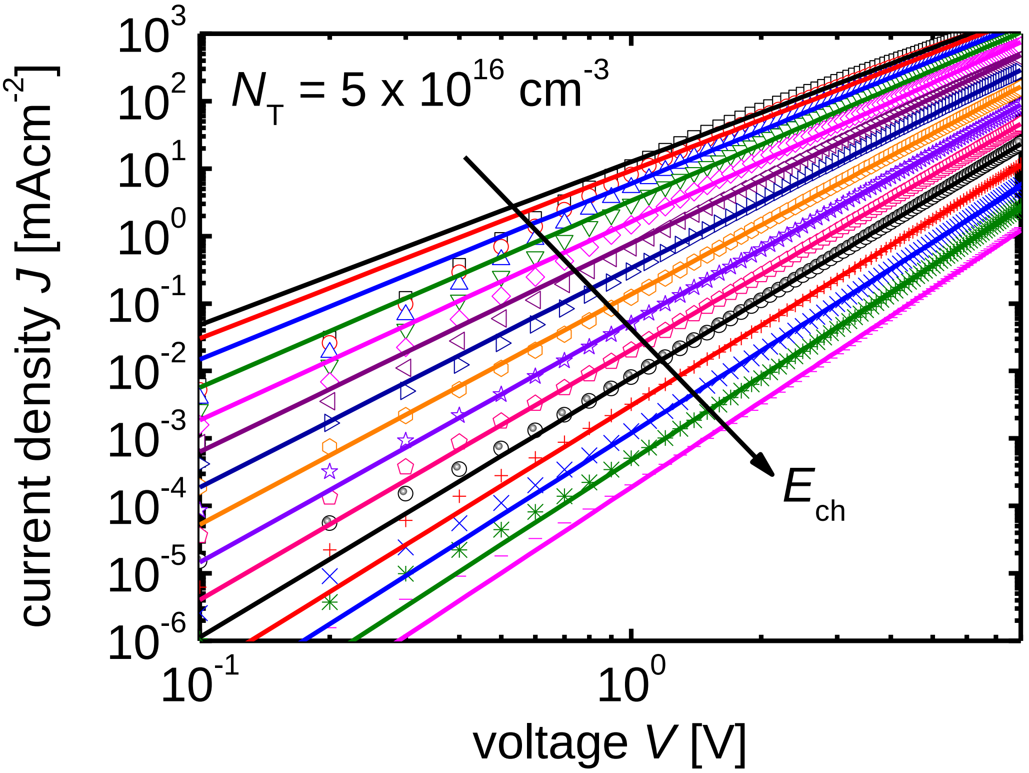

Figure S5: Fits for the case with $N_T = 5 \times 10^{16}$ cm^{-3} for different characteristic tail slopes E_{ch} from 30 meV to 100 meV. Symbols are the simulations with the drift-diffusion solver and lines are the fits with the Mark–Helfrich equation. The fits are done in the voltage range 0.1 V < V < 8 V.

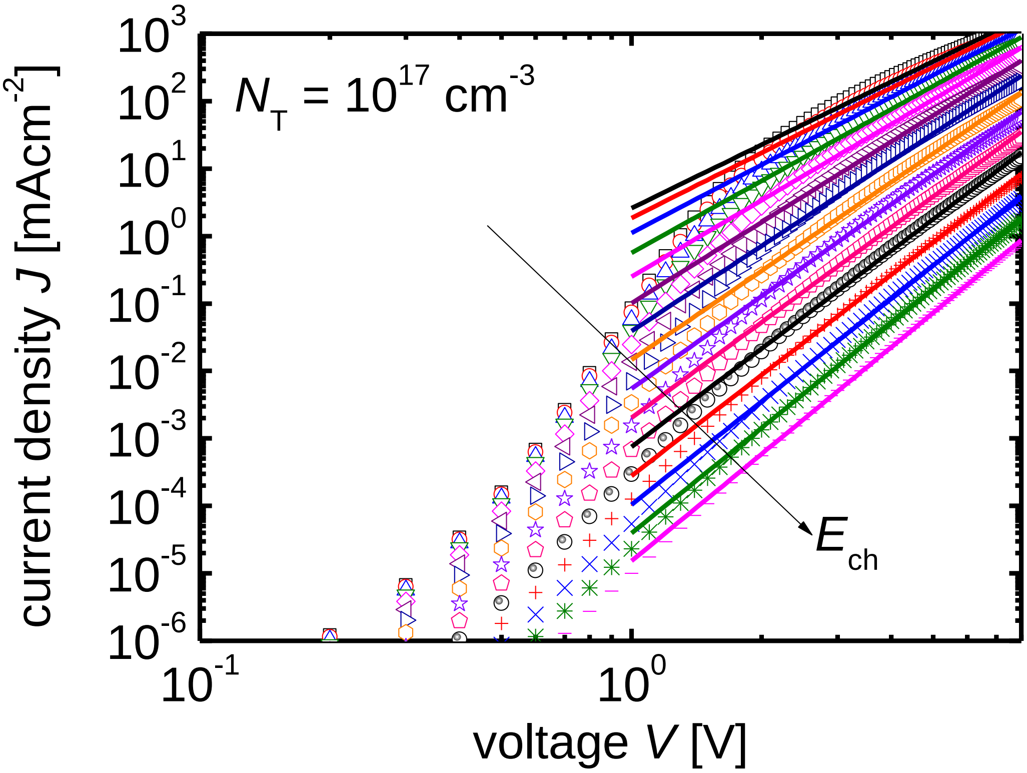

Figure S6: Fits for the case with N_T = 10^{17} cm^{-3} for different characteristic tail slopes E_{ch} from 30 meV to 100 meV. Symbols are the simulations with the drift-diffusion solver and lines are the fits with the Mark-Helfrich equation. The fits are done in the voltage range 1 V < V < 8 V, because the reduced current at low bias (V < 1 V) cannot be fitted with the same relation as the high voltage range (V > 1 V).
